# Transcription Factor-Mediated Generation of Dopaminergic Neurons from Human iPSCs—A Comparison of Methods

**DOI:** 10.3390/cells13121016

**Published:** 2024-06-11

**Authors:** Kirstin O. McDonald, Nikita M. A. Lyons, Luca K. C. Gray, Janet B. Xu, Lucia Schoderboeck, Stephanie M. Hughes, Indranil Basak

**Affiliations:** Department of Biochemistry, Brain Health Research Centre and Genetics Otago, University of Otago, Dunedin 9016, New Zealand

**Keywords:** human-induced pluripotent stem cells, dopaminergic neurons, transcription factors, ASCL1, LMX1A, NURR1, directed differentiation, CRISPR-Cas9, lentivirus transduction

## Abstract

Dopaminergic neurons are the predominant brain cells affected in Parkinson’s disease. With the limited availability of live human brain dopaminergic neurons to study pathological mechanisms of Parkinson’s disease, dopaminergic neurons have been generated from human-skin-cell-derived induced pluripotent stem cells. Originally, induced pluripotent stem-cell-derived dopaminergic neurons were generated using small molecules. These neurons took more than two months to mature. However, the transcription-factor-mediated differentiation of induced pluripotent stem cells has revealed quicker and cheaper methods to generate dopaminergic neurons. In this study, we compared and contrasted three protocols to generate induced pluripotent stem-cell-derived dopaminergic neurons using transcription-factor-mediated directed differentiation. We deviated from the established protocols using lentivirus transduction to stably integrate different transcription factors into the AAVS1 safe harbour locus of induced pluripotent stem cells. We used different media compositions to generate more than 90% of neurons in the culture, out of which more than 85% of the neurons were dopaminergic neurons within three weeks. Therefore, from our comparative study, we reveal that a combination of transcription factors along with small molecule treatment may be required to generate a pure population of human dopaminergic neurons.

## 1. Introduction

Induced pluripotent stem cell (iPSC) research has helped neuroscientists minimise the dependence on transgenic/knockout animal models and human cells like embryonic stem cells or cells derived from biopsies [1,2]. The versatility of iPSCs has been harnessed in other fields of research, such as cardiac and renal diseases [3,4]. Many groups have embarked on differentiating skin fibroblast-derived iPSCs into dopaminergic neurons, from both healthy and Parkinson’s disease-affected individuals, using different approaches such as small molecules, transcriptions factors, and microRNAs [1,5,6,7,8,9,10,11,12,13,14]. The original protocol published more than a decade ago by Kriks et al. [6] used small molecules to generate human dopaminergic neurons from iPSCs; however, the protocol took more than two months to achieve mature human dopaminergic neurons. Other studies [15,16,17] have used modified protocols with small molecules and have obtained purer populations of dopaminergic neurons; however, the time to develop the mature neurons was still more than a month. Since 2011, several researchers have developed protocols for transcription factor-mediated differentiation using various transcription factors [7,8,9,10,11,14], and have successfully generated iPSC-derived human dopaminergic neurons in a relatively short amount of time (less than one month). Theka et al. [5] was among the first to generate iPSC-derived dopaminergic neurons through transcription factor-mediated differentiation, omitting the neural progenitor cell stage. The protocol involved lentivirus transduction of four components: reverse-tetracycline-controlled transactivator (rtTA) and three transcription factors, Ascl1, Nurr1, and Lmx1a. These transcription factors are neurodevelopmental factors, particularly in dopaminergic development. Prior studies [14] have shown that forced expression of these three transcription factors can directly reprogram fibroblasts into functional dopaminergic neurons. Ascl1 is a DNA-binding transcription factor that can access closed chromatin to allow other factors to bind and activate neural pathways [18]. Nurr1 is an orphan nuclear receptor that is critical in the development and maintenance of the dopaminergic neuronal population as it can regulate tyrosine hydrolase expression, dopamine metabolism, terminal differentiation, axon outgrowth, and neuronal patterning and survival [19]. Lmx1a is the LIM homeobox transcription factor that induces msh homeobox 1 (Msh1) that further activates Neurogenin 2 (Ngn2), thereby promoting neuronal differentiation and suppressing alternate ventral cell fate. Lmx1a is expressed in midbrain dopamine progenitor cells and regulates the induction of dopaminergic neurons [20]. Nishimura et al. 2022 [8] used a PiggyBac Transposase Expression Vector system to integrate two transcription factors (ASLC1 and LMX1A) to generate human dopaminergic neurons. In the same year, Sheta et al. [9] used only one transcription factor (NGN2) to differentiate iPSCs into dopaminergic neurons. However, the bottleneck has always been getting a pure population of dopaminergic neurons, something that Zhang et al. [21] and Fernandopulle et al. [22] achieved with human glutamatergic cortical-like neurons. This bottleneck is a hindrance to cell replacement therapy in Parkinson’s disease, where dopaminergic neurons are the most vulnerable neuronal population in the brain and the first ones to degenerate.

In this study, we modified and compared three different protocols using transcription factors to generate human dopaminergic neurons. We stably integrated transcription factors into iPSCs using CRISPR-Cas9 editing and controlled their expression through a tetracycline-inducible promoter. The stable integration enabled us to genetically modify the iPSCs in a one-step protocol and eliminated variation from co-transduction or sequential transduction of different lentiviruses. CRISPR-Cas9 gene editing causes double-strand breaks, which also induces a p53-mediated cell death [23]. To counter this problem, we used a non-integrating dominant negative p53 construct along with the donor DNA (transcription factors), single guide RNAs, and Cas9 protein while generating the iPSC lines stably expressing the transcription factors.

Using different media compositions, we achieved the generation of iPSC-derived human dopaminergic neurons (iDA) from all three protocols within three weeks. However, the percentage of iDAs varied significantly from what has been reported by the respective studies. The modification we applied to the protocol from Sheta et al. yielded the highest percentage of tyrosine hydroxylase and GIRK2-positive dopaminergic neurons in 3 weeks, which were also electrophysiologically active and released dopamine. Our results suggest that a finely optimised combined approach may be required for the generation of pure populations of dopaminergic neurons. Nonetheless, our in vitro model is far from generating dopaminergic neurons for transplantation purposes, particularly due to the expression of transcription factors. However, these iPSC-derived dopaminergic neurons can serve as an excellent complementary model to understand molecular mechanisms and screen for therapeutic candidates for Parkinson’s disease.

## 2. Materials and Methods

### 2.1. Reagents

For the three different protocols to generate iPSC-derived iDAs, the following reagents were used. Media compositions are included in Table S1.

For iPSCs—WTC11 human-induced pluripotent stem cells (gifted by Dr Michael Ward, National Institute of Health (NIH), Bethesda, MD, USA), Matrigel (cat# 354277), Rock Inhibitor (RI) Y-27632 (cat# RDS125410) (both from In Vitro Technologies, Auckland, New Zealand), Essential 8 (E8) medium (cat# A1517001), EDTA (0.5M) pH 8.0 (cat# AM9261), PBS pH 7.4 (cat# 70011044), Accutase (cat# A1110501) (all from ThermoFisher Scientific, Auckland, New Zealand).

For genotyping and polymerase chain reactions—PureLink Genomic DNA kit (cat# K182001, ThermoFisher Scientific, Auckland, New Zealand); Phusion High-Fidelity DNA Polymerase (cat# M0530L, New England Biolabs, Ipswich, MA, USA); and Sanger sequencing at Massey Genome Service, Palmerston North, New Zealand.

For recombinant lentivirus production—HEK293FT cell line (cat# R70007, ThermoFisher Scientific, Auckland, New Zealand); HT1080 cell line (cat# CCL-121, ATCC, Manassas, VA, USA); psPAX2 (cat# 12260, Addgene, Watertown, MA, USA); VSVg (cat# K497500); DMEM, high glucose, pyruvate, no glutamine (cat# 10313021); MEM Non-Essential Amino Acids Solution (100×) (cat# 11140050); L-glutamine (200 mM) (cat# 25030081); sodium pyruvate (100 mM) (cat# 11360070); penicillin–streptomycin (10,000 U/mL) (cat# 15140122); Opti-MEM™ Reduced Serum Medium (cat# 31985070); TrypLE™ Express Enzyme (1×); phenol red (cat# 12605028); Lipofectamine™ 2000 Transfection Reagent (cat# 11668019) (all from ThermoFisher Scientific, Auckland, New Zealand); foetal bovine serum—sterile filtered (cat# FBSF, (Moregate BioTech, Bulimba, QLD, Australia); polybrene (H9-268); poly-L-Lysine hydrochloride (cat# 9404); lactose (cat# MR5245) (all from Merck, Auckland, New Zealand).

For iPSC-derived iDA generation following Theka et al. [5]—Alt-R CRISPR-Cas9 AAVS1 sgRNAs and Alt-R S.p. Hifi Cas9 Nuclease V3 (cat# 104906119) (IDT, Melbourne, VIC, Australia), OptiMEM I reduced serum medium (cat# 31985070), Lipofectamine Stem transfection reagent (cat# STEM00001), DMEM/F12 (cat# 11330032), penicillin–streptomycin (10,000 U/mL) (cat# 15140122), B27 supplement (cat# 17504044) (all from ThermoFisher Scientific, Auckland, New Zealand), puromycin dihydrochloride (cat# P8833), insulin (cat# I9278), transferrin (cat# T8158), sodium selenite (cat# 214485), progesterone (cat# P6149), putrescine (cat# P5780), doxycycline (cat# D9891), paraformaldehyde (cat# P6148), and 4′,6-diamidino-2-phenylindole (DAPI) (D9542) (all from Merck, Auckland, New Zealand). See Table S2 for the antibodies used for immunocytochemistry (ICC). Dopamine release from the iPSC-derived iDAs was measured using the Dopamine ELISA kit (cat# ab285238, Abcam, Melbourne, Australia).

For iPSC-derived iDA generation following Nishimura et al. [8]—essential 6 medium (E6) (cat# A1516401), neurobasal medium (cat# 21103049), B27 supplement minus vitamin A (cat# 12587010), GlutaMax (cat# 35050061) (all from ThermoFisher Scientific, Auckland, New Zealand), puromycin dihydrochloride (cat# P8833), doxycycline (cat# D9891), dibutyryl cyclic AMP (dbcAMP; cat# D0627) (all from Merck, Auckland, New Zealand), DAPT (cat# 2088055, Peprotech/Lonza, Sydney, Australia), brain-derived neurotrophic factor (BDNF; cat# RDS248BDB050, In Vitro Technologies, Auckland, New Zealand), glial cell derived neurotrophic factor (GDNF; cat# 450-01), and ascorbic acid (cat# 5088177) (both from PeproTech, Rocky Hill, NJ, USA). See Table S2 for the antibodies used for ICC. Dopamine release from the iPSC-derived iDAs was measured using the Dopamine ELISA kit (cat# ab285238, Abcam, Melbourne, Australia).

For iPSC-derived iDA generation following Sheta et al. [9]—DMEM/F12 (cat# 11330032), L-glutamine (cat# 25030081), non-essential amino acids (NEAA; cat# 11140050), N-2 supplement (cat# 17502048), laminin (cat# 23017015), B27 supplement (cat# 17504044) (all from ThermoFisher Scientific, Auckland, New Zealand), poly-L-ornithine (cat# P3655), cytosine β-D-arabinofuranoside hydrochloride (Ara-C) (cat# C6645) (both from Merck, Auckland, New Zealand), BDNF (cat# RDS248BDB050), NT-3 (cat# RDS267N3025) (both from In Vitro Technologies, Auckland, New Zealand), BrainPhys Neuronal Media (cat# 05790), STEMdiff Midbrain Neuron Differentiation kit (cat# 100-0038), STEMdiff Midbrain Neuron Maturation kit (cat# 100-0041), human recombinant shh (C24II) (cat# 78065.1) (all from STEMCELL Technologies, Tullamarine, VIC, Australia). See Table S2 for the antibodies used for ICC. Dopamine release from the iPSC-derived iDAs was measured using the Dopamine ELISA kit (cat# ab285238, Abcam, Melbourne, Australia).

### 2.2. iPSC-Derived Dopaminergic Neuron Generation following Theka et al.

Theka et al. [5] presented the first data showing that the expression of three transcription factors, ASCL1, NURR1, and LMX1A, can drive iPSCs towards neuronal dopaminergic differentiation (iDAs). The authors used lentiviral methods to induce the expression of the three transcription factors into iPSCs followed by the use of a neuronal-inducing media for 7–18 days to generate dopaminergic neurons. We used the following two different methods to integrate the transcription factors into the iPSCs.

#### 2.2.1. Overexpression of Transcription Factors Using Recombinant Lentiviruses

The transcription factors and the reverse tetracycline transactivator (rtTA) were obtained from Addgene (Tet-O-Fuw-Ascl1 Addgene plasmid #27150, pLV.PGK.mLmx1a Addgene plasmid #33013, Nurr1 Addgene plasmid #35000, FUW-M2rtTA Addgene plasmid #20342). The cassette with the three human transcription factors hALAN—human ASCL1, LMX1A, and NURR1—was synthesised by GeneArt Gene Synthesis Service (ThermoFisher Scientific, Auckland, New Zealand) and cloned into a lentiviral plasmid with mNeonGreen as a marker driven by EF1a promoter (Figure S1A). The rtTA was cloned into another lentiviral plasmid with blue fluorescence protein (BFP) as a marker driven by EF1a promoter (Figure S1A). Lentiviral generation was performed following our established protocol [24]. iPSCs were transduced with the transcription factor lentivirus and the rtTA lentivirus at a multiplicity of infection (MOI) of 1, followed by fluorescence-activated cell sorting (FACS) [25] for mNeonGreen and BFP (Figure S1B). The double-positive (mNeonGreen and BFP) enriched iPSCs were expanded and cryopreserved for downstream differentiation into iDAs.

#### 2.2.2. Integration of Transcription Factors Using the CRISPR-Cas9 Approach

The synthesised hALAN was cloned into a donor construct to introduce the hALAN into the AAVS1 safe harbour site for iPSC differentiation (Addgene plasmid #105840). The resulting plasmid expresses hALAN, rtTA, and mApple. To stably integrate the hALAN cassette into the AAVS1 site and generate iPSCs expressing hALAN for iDA differentiation, a workflow outlined in Figure S2A–D was followed. iPSCs were transfected with 500 ng of the hALAN plasmid, 3 pmol of 2 sgRNAs against AAVS1 (Table S3 for sgRNA sequences), 3 pmol of Alt-R HiFi Cas9 enzyme, and 100 ng of pCE-mp53DD (dominant-negative p53; addgene plasmid # 41856) using Lipofectamine Stem following the manufacturer’s protocol. The day following transfection, transfected iPSCs were passaged with Accutase and plated for expansion. The mApple-positive iPSCs were isolated by FACS, and the sorted iPSCs were expanded, cryopreserved, and plated for clonal outgrowth at 50% serial dilutions. Individual colonies were picked manually, expanded, and treated with 1 μg/mL puromycin followed by genotyping to ensure the integration of hALAN.

#### 2.2.3. Genotyping of iPSCs with Integrated hALAN

Genomic DNA was extracted from the mApple-sorted iPSC colonies using a PureLink Genomic DNA kit following the manufacturer’s protocol. Genomic DNA was quantified using Nanodrop and used for PCR with Phusion polymerase following the manufacturer’s protocol to detect the transcription factors. Primers used for genotyping are included in Table S3.

#### 2.2.4. Differentiation of iPSCs into iDAs

Following the original protocol published by Theka et al. [5], the mApple-sorted iPSCs with the three transcription factors integrated at the AAVS1 site were differentiated using the media composition outlined in Figure S2E (and Table S1). iPSCs were passaged with Accutase and plated on a Matrigel-coated plate in E8+RI. After two days, the E8 media was replaced with neuronal inducing medium containing DMEM/F12, 25 μg mL^−1^ insulin, 50 μg/mL transferrin, 30 nM sodium selenite, 20 nM progesterone, and 100 nM putrescine and doxycycline (2 μg/mL). After 7 days of differentiation, B27 was added to the aforementioned media. The medium was changed every 2–3 days, and the cells were maintained in culture medium for 21 and 28 days for the maturation of the iDAs followed by assessment by ICC.

### 2.3. iPSC-Derived Dopaminergic Neuron Generation following Nishimura et al.

The following protocol was adopted from Nishimura et al. [8] to differentiate the hALAN-integrated mApple-sorted iPSCs into iDAs (Figure S3A and Table S1). iPSCs were passaged with Accutase and plated on a Matrigel-coated plate in E8+RI. Two days after plating, the E8 media was replaced with Essential 6 medium (E6) supplemented with 5.0 mg/mL DOX and 1.0 μg/mL puromycin for the first 3 days. After 3 days, the E6 medium was changed to Neurobasal medium supplemented with B27 supplement vitamin A minus, GlutaMax, 5.0 mg/mL DOX, and 1.0 mg/mL puromycin and maintained for the next 4 days. After three days in E6 media and four days in Neurobasal media, the neuronal-inducing media was supplemented with 10 μM DAPT for the next 3 days followed by supplementing with 20 ng/mL BDNF, 10 ng/mL GDNF, 200 mM ascorbic acid, and 500 mM dbcAMP and continued until day 21. On day 21, the iDAs were used for ICC.

### 2.4. iPSC-Derived Dopaminergic Neuron Generation following Sheta et al.

The following protocol was adopted from Sheta et al. [9] and Liu et al. [26] to differentiate the iPSCs into iDAs. With an established protocol to generate iPSC-derived cortical neurons (i^3^Ns) [22,27], our i^3^N protocol was combined with the iDA protocol from Sheta et al. to modify the media compositions and generate purer populations of iDAs. For this protocol, the hALAN iPSCs were not used; rather, iPSCs with integrated NGN2 transcription factor were used [21,22,27]. These NGN2-iPSCs express NGN2 under the control of a doxycycline-inducible promoter, as developed by Fernandopulle et al. [22]. The NGN2-integrated iPSCs were initially plated on a Matrigel-coated plate in E8+RI, and after two days of culture (ensuring colony formation), the iPSCs were passaged with Accutase to obtain a single-cell suspension. The single-cell suspension of iPSCs was re-plated on a Matrigel-coated plate in warm induction media (Table S1) supplemented with doxycycline (2 μg/mL) and RI (1 μg/mL). For the next two days, the induction media was replaced with more warm induction media supplemented with doxycycline (2 μg/mL). After three days of doxycycline-mediated induction of NGN2 expression, the induced iPSCs were harvested using Accutase and re-plated on poly-L-ornithine (PLO, 100 μg/mL final concentration) and laminin (10 µg/mL final concentration)-coated plates in cortical neuron media (Table S1). Two days after plating the induced iPSCs in cortical neuron media, the cortical neuron media was completely replaced with STEMDiff Midbrain Differentiation Media (Table S1) supplemented with cytosine β-D-arabinofuranoside hydrochloride (Ara-C) at 2 µM (to inhibit the proliferation of any remaining dividing cells) and doxycycline (2 μg/mL). The differentiation media was completely replaced with fresh iDA differentiation media supplemented with Ara-C at 2 µM and doxycycline (2 μg/mL) for the next two days followed by half media changes (iDA-differentiation-media-supplemented doxycycline (2 μg/mL)) for another four days. Finally, after eight days of differentiation, the media was completely replaced with fresh iDA maturation media (Table S1) supplemented with doxycycline (2 μg/mL) on the ninth day. Starting from day 10 and every two days, half of the media was replaced with fresh iDA maturation media supplemented with doxycycline (2 μg/mL) for 22 and 26 days for the maturation of the iDAs followed by assessment by ICC.

### 2.5. Immunocytochemistry and Calcium Signalling on iPSC-Derived iDAs

The iPSC-derived iDAs from all three protocols were used for ICC to ascertain the percentage of dopaminergic neurons being generated. ICC was performed following our previously established protocol [27]. Briefly, the iPSC-derived iDAs were maintained for 3–4 weeks in different media conditions, as outlined in the previous three sections, and were fixed using 4% paraformaldehyde in PBS. The neurons were stained with antibodies against TUJ1 (neuron-specific class III beta-tubulin), MAP2 (dendritic neuronal marker), tyrosine hydroxylase (dopaminergic neuronal marker), GIRK2 (G-protein-regulated inward-rectifier potassium channel 2, neuronal marker characteristic of dopaminergic neurons from substantia nigra of human brain), and LMX1A and NURR1 (transcription factors used to generate iPSC-derived dopaminergic neurons) (Table S2). Alexa Fluor-labelled secondary antibodies (ThermoFisher) were used as per Table S2, followed by staining with 4′,6-diamidino-2-phenylindole (DAPI). The stained neurons were imaged on a Nikon Eclipse Ti2 epi-fluorescence microscope (Nikon, Tochigi, Japan) and Olympus FV3000 confocal microscope (Olympus, Tokyo, Japan). For calcium imaging, i^3^Ns and iDAs were transduced with lentivirus expressing GCaMP7s driven by the Synapsin promoter (pCDH.rSyn.jGCaMP7s—cloned from pGP-AAV-syn-jGCaMP7s-WPRE, Addgene plasmid #104487) two days before live cell imaging. Using an Olympus FV3000 confocal microscope (Olympus, Tokyo, Japan) with a 37 °C heated chamber, 100 frames were captured per field (n ≥ 3 fields per condition) with an interval of 2 s to obtain time-lapse videos of calcium signalling in the i^3^Ns and the iDAs. The videos were analysed following Muto et al. [28].

### 2.6. Dopamine Release Assay on iPSC-Derived iDAs

To test whether the in vitro models of iPSC-derived iDAs from all three protocols release dopamine, we tested the media from the cell cultures for dopamine content using the Dopamine ELISA kit following the manufacturer’s protocol. Briefly, the iDAs were maintained for 21 days in their respective media (as mentioned in Section 2.1), and on day 21, media were collected for the ELISA from iDAs with no KCl treatment and iDAs treated with 56 mM KCl for 15 min (following [29]). The media was centrifuged to pellet any cells, and the supernatant was added to the ELISA plate, followed by processing according to the manufacturer’s protocol. After the indicated incubations, absorbance was measured at 450 nm. The absorbance values from the standards were used to plot a standard curve, and the standard curve equation was used to calculate the dopamine released from the iDAs using the absorbance measured from the iDA media samples. The concentrations of dopamine obtained from the iDA media samples were normalised to the number of cells plated for every protocol.

### 2.7. Image Analysis and Statistical Analysis

Images acquired on the Nikon and Olympus microscopes were analysed using ImageJ version 1.53 (NIH, Bethesda, MD, USA). For DAPI counts, the following workflow was used: Open (image) > Image > Adjust Threshold (set threshold to separate the individual DAPI-positive nuclei; in this case, it was set between 0 and 6600) > Process > Binary > Make binary > Process > Binary > Outline > Measure > Analyse Particle > Set Size (0.50-infinity) and Circularity (0.00–1.00) > Show Outlines > Note the ‘Count’ from the Summary window. For TUJ1, MAP2, and TH counts, the neuronal cell bodies were counted showing positive staining using a similar workflow as above. The counts for TUJ1, MAP2, and TH-positive cells were normalised by the counts for DAPI followed by calculating the percentage of TUJ1, MAP2, and TH-positive cells on MS Excel. The percentage positive values were imported onto GraphPad Prism (GraphPad, San Diego, CA, USA) and tested for normal distribution, and unpaired two-tailed Student’s t-tests were performed to assess statistical significance, reported as *p*-values. Data are presented as mean ± standard error of mean (SEM). * indicates *p* < 0.05, ** indicates *p* < 0.01, *** indicates *p* < 0.001.

## 3. Results

### 3.1. Targeted and Stable Integration in iPSCs Maintained Transcription Factor Expression Better than Lentiviral Transduction

**Figure 1:**
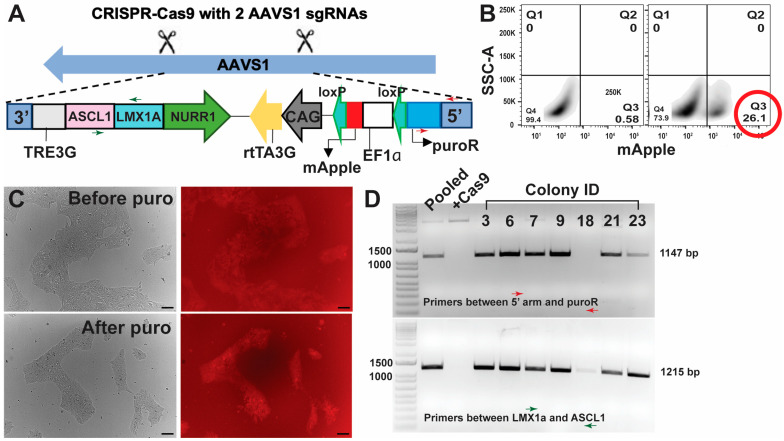
Integration of transcription factors and selection of iPSC colonies. (**A**) Using CRISPR-Cas9, the cassette containing the 3 human transcription factors (ASCL1, LMX1A, and NURR1), the puroR gene, and the mApple selection marker were integrated into the AAVS1 safe harbour locus of iPSCs. (**B**) After the CRISPR-Cas9 edit of iPSCs, FACS was used to isolate the mApple-positive iPSCs (>25%). The left panel shows the FACS gating of the iPSCs with no mApple and the right panel shows the percent mApple-positive iPSCs based on the gating from the left panel. FlowJo (v10.10.0) was used to generate the FACS images. (**C**) The mApple-positive sorted iPSCs were used to pick colonies manually, which were treated with puromycin to obtain a purer population of mApple-positive iPSCs. Scale bar 100 μm. (**D**) The FACS and puromycin-treated iPSC colonies were genotyped using PCR, amplifying a partial region of the 5′ arm and the puroR (red arrows) and a partial region of the LMX1a and the ASCL1 transcription factors (green arrows). Also refer to (**A**) for the position of the primers indicated by the red and the green arrows. DNA gel showing ladder in lane 1, pooled iPSCs (before colony picking) in lane 2, Cas9-only iPSCs (no hALAN integration) in lane 3, and colonies with positive/negative PCR bands in the following lanes. The numbers above the lanes represent colony ID numbers.

Post-transduction of the two lentiviruses (Figure S1A) in the iPSCs, following Theka et al. [5], the iPSCs were sorted for BFP and mNeonGreen (Figure S1B). The percentage of double-positive iPSCs (BFP+, mNeonGreen +) was less than 15% (Figure S1B, second plot), and the double-positive iPSCs were expanded (Figure S1Ci) for differentiation. However, with passaging, the double-positive iPSCs started losing the mNeonGreen fluorescence (Figure S1Cii). Although whether the hALAN construct was lost from the iPSCs was not verified, with the recent success of stably integrating transcription factors into iPSC safe harbour loci [22], an alternative method (Figure S2) to integrate hALAN was adopted. The alternative method involved transfection of the hALAN cassette (Figure S2A), FACS (Figure S2B), serial dilution, manual picking and puromycin selection (Figure S2C), and validation of clones by genotyping (Figure S2D). Using CRISPR-Cas9, the hALAN construct, along with the mApple, puroR, and rtTA, were integrated into the AAVS1 safe harbour locus of iPSCs (Figure 1A), which yielded mApple+ iPSCs (Figure 1B, second plot). The bulk FACS of mApple yielded > 25% mApple+ iPSCs (Figure 1B, second plot), which were expanded, cryopreserved, and later re-plated to pick colonies manually. The picked colonies were treated with puromycin to obtain a purer population of mApple+ iPSCs (Figure 1C), followed by genotyping by PCR (Figure 1D) and sequencing. Using primers spanning ASCL1 + LMX1A and 5′ AAVS1 arm + PuroR, six colonies were identified that had hALAN integrated into the AAVS1 site. Colonies #6 and #9 were used for the differentiation protocol.

### 3.2. Differentiation following Theka et al. and Nishimura et al.

**Figure 2:**
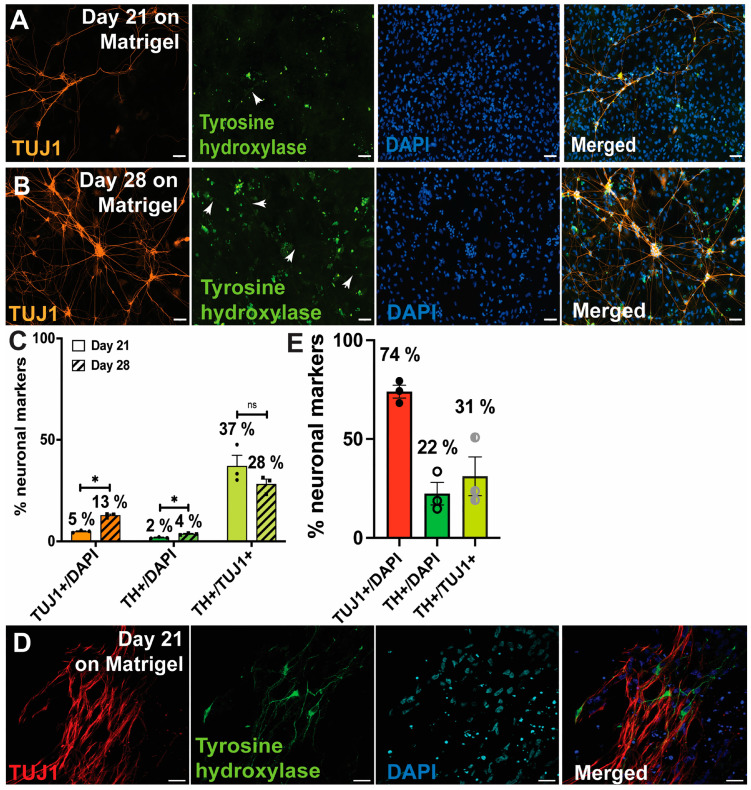
Differentiation of iPSCs into iDAs following Theka et al. and Nishimura et al. (**A**,**B**) iPSC-derived day 21 and day 28 iDAs, respectively, following Theka et al. Cells were stained with TUJ1 (class III β tubulin, neuronal marker), tyrosine hydroxylase (dopaminergic neuronal marker), and DAPI (nuclear stain). Scale bar 100 μm. (**C**) Quantification of TUJ1+ (over DAPI+), TH+ (over DAPI+), and TUJ1+ cells that were also TH+ in day 21 and day 28 iDAs following Theka et al. Percentages show the proportion of cells positive for respective markers. (**D**) iPSC-derived day 21 iDAs, following Nishimura et al. Cells were stained with TUJ1 (class III β tubulin neuronal marker), tyrosine hydroxylase (dopaminergic neuronal marker), and DAPI (nuclear stain). Scale bar 20 μm. (**E**) Quantification of TUJ1+ (over DAPI+), TH+ (over DAPI+), and TUJ1+ cells that were also TH+ in day 21 iDAs following Nishimura et al. Percentages show the proportion of cells positive for respective markers. Data are presented as mean ± standard error of mean. * *p* < 0.05, ns is not significant.

Figure S2E summarises the differentiation protocol with different media compositions over 3 weeks adapted from Theka et al. Similar to the original paper, our ICC experiments after 3 weeks of differentiation showed the generation of TUJ1+ neurons. However, day 21 cells showed only 5% TUJ1+ neurons (Figure 2A,C) and only 2% TH staining (Figure 2A, white arrow showing weak TH staining in neuronal processes). When the differentiation was extended to 28 days, the percentage of TUJ1+ cells improved significantly to 13% (Figure 2B,C), but still showed low TH staining (4%) (Figure 2B, white arrow showing weak TH staining in neuronal processes). TH staining was brighter in the cell bodies compared to the neuronal processes (Figure 2A,B). Combining the percentage of TH+ and TUJ1+ cells, day 21 cells showed 37% of the TUJ1+ cells were also TH+, whereas day 28 cells showed 28% of the TUJ1+ cells were also TH+ (Figure 2C). There was no significant difference between day 21 and day 28 percentage of TH+/TUJ1+ neurons (Figure 2C). Our i^3^N protocol [27] uses PLO instead of Matrigel to coat cell culture plates for plating and differentiating neurons. Therefore, as an alternative coating surface, PLO-coated wells were also used in addition to Matrigel-coated wells. As iPSCs would not survive on PLO-coated wells, the differentiation was started following Theka et al. [5], and after 7 days of differentiation (before switching to B27 media on Day 8), pre-differentiated cells were lifted with Accutase and plated on PLO-coated wells and maintained until day 21 or 28. Our modified protocol on PLO-coated wells did not improve the generation of TUJ1+ neurons (Figure S2F,G). Therefore, we further modified the existing protocol by incorporating new media compositions (Figure S3A) following Nishimura et al. [8]. ICC on the iDAs differentiated from hALAN iPSCs using the Nishimura protocol resulted in improved generation of TUJ1+ neurons (74%) (Figure 2D,E). More than 22% of the cells also stained positive for TH (Figure 2D,E). However, only 31.2% of TUJ1+ neurons were also TH+, indicating that the population of neurons being generated from this protocol are predominantly not TH+, i.e., not dopaminergic neurons (Figure 2D,E and Figure S3B). GIRK2 is a marker characteristic of dopaminergic neurons from the substantia nigra and the central tegmental area [30], and hence we also tested for GIRK2 expression in the iPSC-derived iDAs alongside expression of the transcription factors LMX1A and NURR1 that are involved in dopaminergic neurogenesis (also present in the hALAN cassette). Interestingly, most cells showed GIRK2+ staining (compare overlap of GIRK2 and DAPI in Figure S3C), but not all cells showed a distinct expression of LMX1A and NURR1 (compare overlap of LMX1A, NURR1, and DAPI in Figure S3D). Therefore, although the Nishimura protocol showed a better yield of neurons, particularly dopaminergic neurons, the protocol produced an impure population of dopaminergic neurons with the possible inclusion of other types of unclassified cells.

### 3.3. Differentiation following Sheta et al.

**Figure 3:**
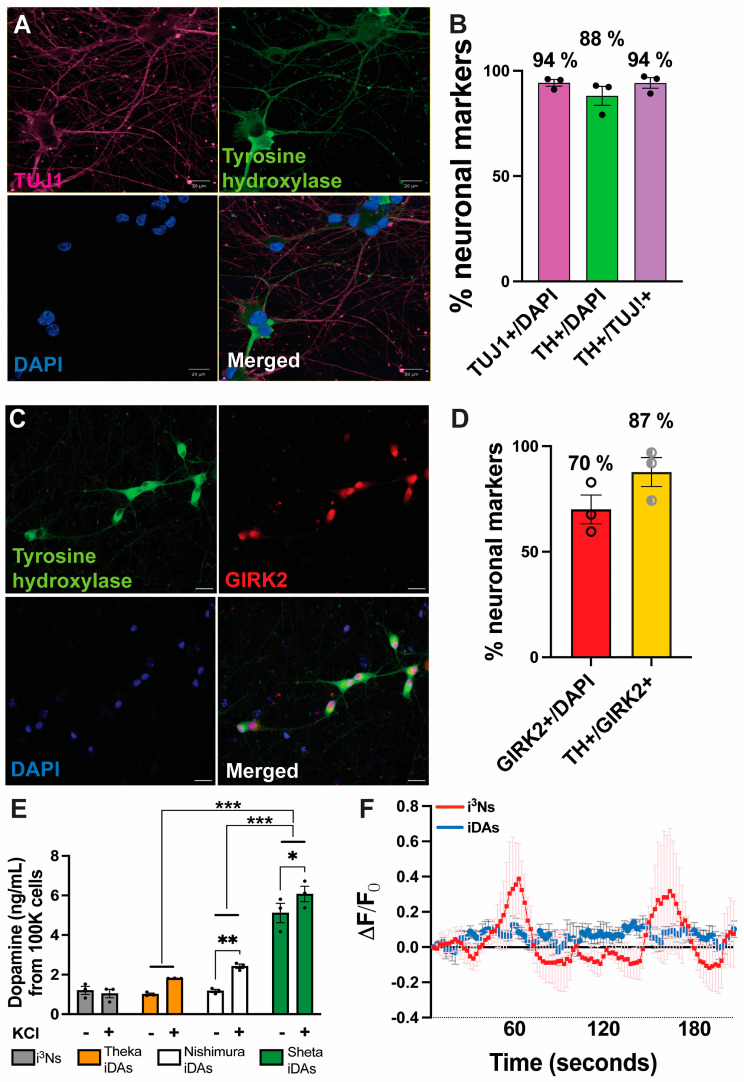
Differentiation of iPSCs into iDAs following Sheta et al. (**A**) iPSC-derived day 21 iDAs were stained with TUJ1 (class III β tubulin, neuronal marker), tyrosine hydroxylase (dopaminergic neuronal marker), and DAPI (nuclear stain). (**B**) Quantification of TUJ1+ (over DAPI+), TH+ (over DAPI+), and TUJ1+ cells that were also TH+ cells in day 21 iDAs following Sheta et al. Percentages show the proportion of cells positive for respective markers. (**C**) Day 21 iDAs were stained with tyrosine hydroxylase (dopaminergic neuronal marker), GIRK2 (G protein-activated inward rectifier potassium channel 2, dopaminergic neuronal marker), and DAPI (nuclear stain). (**D**) Quantification of GIRK2+ (over DAPI+), and GIRK2+ cells that were also TH+ in day 21 iDAs following Sheta et al. Percentages show the proportion of cells positive for respective markers. (**E**) iDAs derived from iPSCs following Theka et al., Nishimura et al., and Sheta et al. showed differential dopamine release in the presence and absence of KCl induction. i^3^Ns were used as a negative control. (**F**) Quantification of change of fluorescence (∆F/F0) over time from calcium signalling experiments in i^3^Ns and iDAs. Data are presented as mean ± standard error of mean. * *p* < 0.05, ** *p* < 0.01, *** *p* < 0.001. Scale bar 20 μm.

Figure S4A summarises the differentiation protocol with different media compositions over three weeks following Sheta et al. [9]. The original protocol was modified to minimise the number of undifferentiated cells. By incorporating an induction step (from Fernandopulle et al. [22]), the iPSCs were pre-differentiated for 3 days before re-plating them at the desired number for downstream analysis (Figure S4A). Twenty-one days post-differentiation, 94% of the cells were TUJ1+, 88% of the cells were TH+, and 94% of the TUJ1+ cells were TH+ (Figure 3A,B). We also stained for vGLUT1 and found that there was no staining for vGLUT1 (glutamatergic neuronal marker, Figure S4B). This is the first time, to our knowledge, that over 85% of dopaminergic neurons have been obtained from iPSCs through transcription-factor-mediated differentiation. Co-staining for TH and GIRK2 showed that 87% of the GIRK2+ were also TH+ (Figure 3C,D). As a negative control, some pre-differentiated cells (gone through the induction step) were plated in cortical neuron media (Table S1) instead of STEMdiff media (Table S1), which stained positive for vGLUT1 and MAP2, but not for TH (Figure S4C). Therefore, combining Fernandopulle and Sheta protocols yielded more than 88% of TH+/TUJ1+ dopaminergic neurons, and 87% of the neurons were positive for both the dopaminergic markers TH and GIRK2.

To confirm the identity of the dopaminergic neurons, we tested dopamine release in the media from the iDAs. iDAs derived following Sheta et al. showed the highest release of dopamine (5.1 ng/mL (equivalent to 33 nmoles/L of dopamine) from 100,000 cells), which was significantly different from the iDAs derived following Theka et al. and Nishimura et al. (Figure 3E). Induction of the iDAs with KCl showed a significant increase in dopamine release from iDAs generated following Nishimura et al. (2.4 ng/mL or 16 nmoles/L) and Sheta et al. (6 ng/mL or 40 nmoles/L) (Figure 3E). Furthermore, to test whether the iDAs are functional like the i^3^Ns [31], we expressed GCaMP7s calcium sensor via lentiviral transduction in three-week-old iDAs and i^3^Ns and recorded changes in calcium signalling as a surrogate for endogenous, non-stimulated electrophysiological properties. From our GCaMP7s experiment, it was observed that the iDAs were electrophysiologically active (Video S1A), similar to what was shown by Mahajani et al. [11], and fire less spontaneously than the i^3^Ns (Video S1B, Figure 3F).

## 4. Discussion

The goal of this study was to generate a pure population of dopaminergic neurons from induced pluripotent stem cells using transcription-factor-mediated differentiation. A purer population of dopaminergic neurons will allow for investigations of Parkinson’s disease-associated genes and pathologies, specifically in affected dopaminergic neurons. In Parkinson’s disease, a major limitation in finding a cure is that by the time of death of the patient, 60–80% of the dopaminergic neurons are dead [32]. Therefore, it is impossible to trace back to when the dopaminergic neurons started dying. To find an early intervention to stop or halt the death of the dopaminergic neurons, we need a way of studying early mechanisms leading to death, which cannot be done in post-mortem human brain tissues. This is where a pure and functional population of iPSC-derived human dopaminergic neurons will serve as a useful model. We modified three published protocols and compared the yield of dopaminergic neurons (Table S4). From our experiments, we confirm that all three protocols can generate human dopaminergic neurons; however, the yield varied considerably from what has been reported in the published protocols. We made modifications to the original protocols following Fernandopulle et al. [22], for example, integration of the transcription factors in the AAVS1 safe harbour locus of iPSCs instead of using lentiviral transduction, as well as using human transcription factors instead of mouse transcription factors to ensure a purer population of human dopaminergic neurons. The modifications made in this study could have led to purer differentiation of iPSCs into dopaminergic neuron lineage only, but they may have compromised the yield of dopaminergic neurons, particularly while following Theka et al. [5] and Nishimura et al. [8]. The limitation stated by Sheta et al. in their protocol [33] for using lentiviral transduction was also addressed in our study by the directed and stable integration of NGN2 into the AAVS1 safe harbour locus of iPSCs. The induction step in our protocol (prior to the differentiation stage) enabled us to include a pause-step where the NGN2-induced partially differentiated cells can be cryopreserved and re-plated later. This step perhaps made the biggest difference between the three protocols. The cells plated following protocols established by Theka et al. and Nishimura et al. retained their non-neuronal morphology until a certain time point, after which the iDA generation and maturation started. In contrast, our inclusion of the induction step in the protocol established by Sheta et al. perhaps ensured a higher percentage of neuron-like cells and excluded any undifferentiated iPSCs, which was also demonstrated by Fernandopulle et al. [22]. The inclusion of Ara-C from Sheta et al. [9,33] also ensures that the cell population for differentiation does not include any undifferentiated and dividing iPSCs. These modifications to the protocol published by Sheta et al. [9] produced a better yield (>85% TH positive and >90% TH and GIRK2 positive) of iPSC-derived dopaminergic neurons compared to previously published protocols [5,6,7,8,9,10,11,12,15,16,17] in only three weeks. Furthermore, we tested dopamine release from the iDAs, which revealed that iDAs generated following Sheta et al. released dopamine that was higher (33–40 nmoles/L) than what was obtained from iPSC-derived dopaminergic neurons generated by Powell et al. [34] and Theka et al. [5]. Although our i^3^Ns also showed some dopamine release in the media, we believe that such low readings are not real and rather represent background levels. Two recently published protocols [35,36], using small molecules, succeeded in obtaining pure populations of dopaminergic neurons (>80%) after more than a month, which were suitable for transplantation purposes. Therefore, perhaps combining the small molecule approach with transcription factor(s) will allow us to get close to 100% yield of pure dopaminergic neurons. Finally, to test whether that the iDAs are electrophysiologically active, our GCaMP7s calcium signalling experiment showed changes in calcium flux without any stimulation.

Fernandopulle et al. [22] discussed that integration of NGN2 transcription factor at the AAVS1 site in iPSCs did not alter any gene expression or function, i.e., did not show any off-target effect. However, caution must be taken when using these cells for transplantation approaches as more in vivo studies might reveal the long-term effect of NGN2 integration. Therefore, further multi-omics analyses and phenotypic assessment must be undertaken before using these cells for transplantation experiments. Nonetheless, our iDA model might serve as an excellent in vitro model to study molecular mechanisms and pathways and to screen for therapeutic candidates in Parkinson’s disease research.

Obtaining a pure population of dopaminergic neurons is a pre-requisite for cell replacement therapy in Parkinson’s disease. Additionally, a pure dopaminergic neuron population would help to study any Parkinson’s disease-associated gene or cellular pathology in the population of neurons that are specifically affected by the disease. However, in the human brain, these dopaminergic neurons do not function alone. The neurons are supported by other brain cells, such as astrocytes and microglia. As seen from our study, the percentage of TH-positive neurons decreased over time, and Otero et al. [37] showed that healthy astrocytes helped the survival of the dopaminergic neurons. Therefore, in future experiments, combining a co-culture of astrocytes and dopaminergic neurons may facilitate better survival of the dopaminergic neurons and offer a more accurate model to study Parkinson’s disease-associated genes and pathologies. Co-culturing with other brain cells may be beneficial for the health of the dopaminergic neurons but will lead to an impure population of dopaminergic neurons. Therefore, once the dopaminergic neurons are mature, healthy, and functional in the co-culture system, they would need sorting (with a dopaminergic neuronal marker) to obtain a pure population. However, sorting is often harsh on the neurons and leads to cell death. Hence, maintaining a high quantity and high quality of dopaminergic neurons is one of the limitations of studies involving the generation of iPSC-derived dopaminergic neurons. As transplantation requires large numbers of pure, healthy, and functional dopaminergic neurons, more optimisation involving co-culture is still required to produce transplantation-grade human dopaminergic neurons.

## Data Availability

All Supplementary Materials include all the data generated in this study leading to the manuscript.

## Supplementary Materials

The following supporting information can be downloaded at https://www.mdpi.com/article/10.3390/cells13121016/s1. Figure S1. Lentiviral transduction of human transcription factors in iPSCs. Figure S2. Workflow to generate iPSC-derived iDAs following Theka et al. and Nishimura et al. Figure S3. Differentiation of iPSCs into iDAs following Nishimura et al. Figure S4. Modified protocol to differentiate iPSCs into iDAs following Sheta et al. Table S1. Media composition. Table S2: Antibodies. Table S3: Sequences. Table S4: iDA comparison. Video S1A: Calcium imaging in iPSC-derived iDAs. Video S1B: Calcium imaging in iPSC-derived i3Ns.
