# Supplementary figures and images for: Transcription Factor-Mediated Generation of Dopaminergic Neurons from Human iPSCs—A Comparison of Methods
Source: Cells. 2024 Jun 11;13(12):1016. doi: 10.3390/cells13121016 (PMC11201854; doi:10.3390/cells13121016)

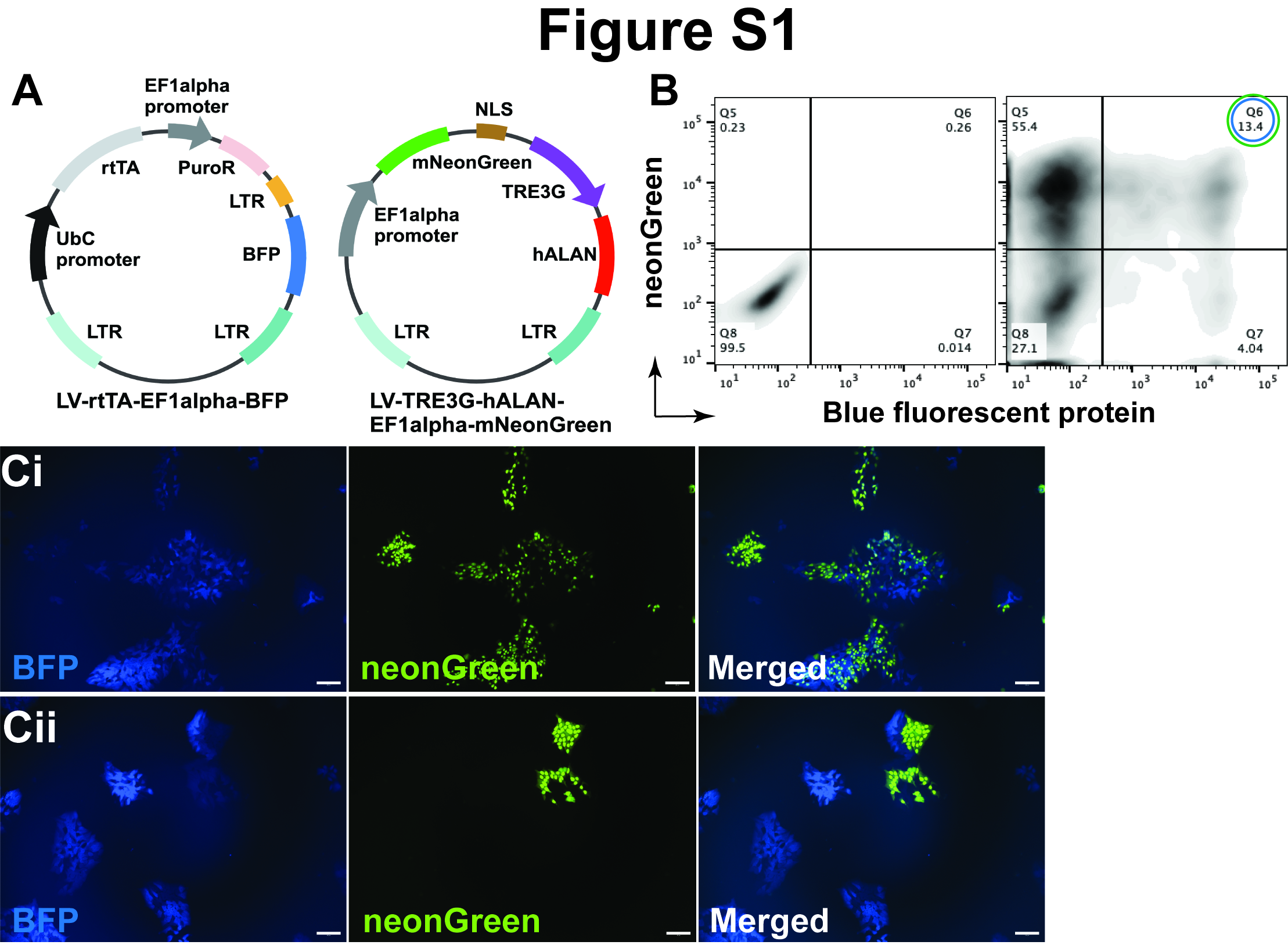

Supplement: Supplementary file 1 [file cells-13-01016-s001.zip › Figure S1.tif]

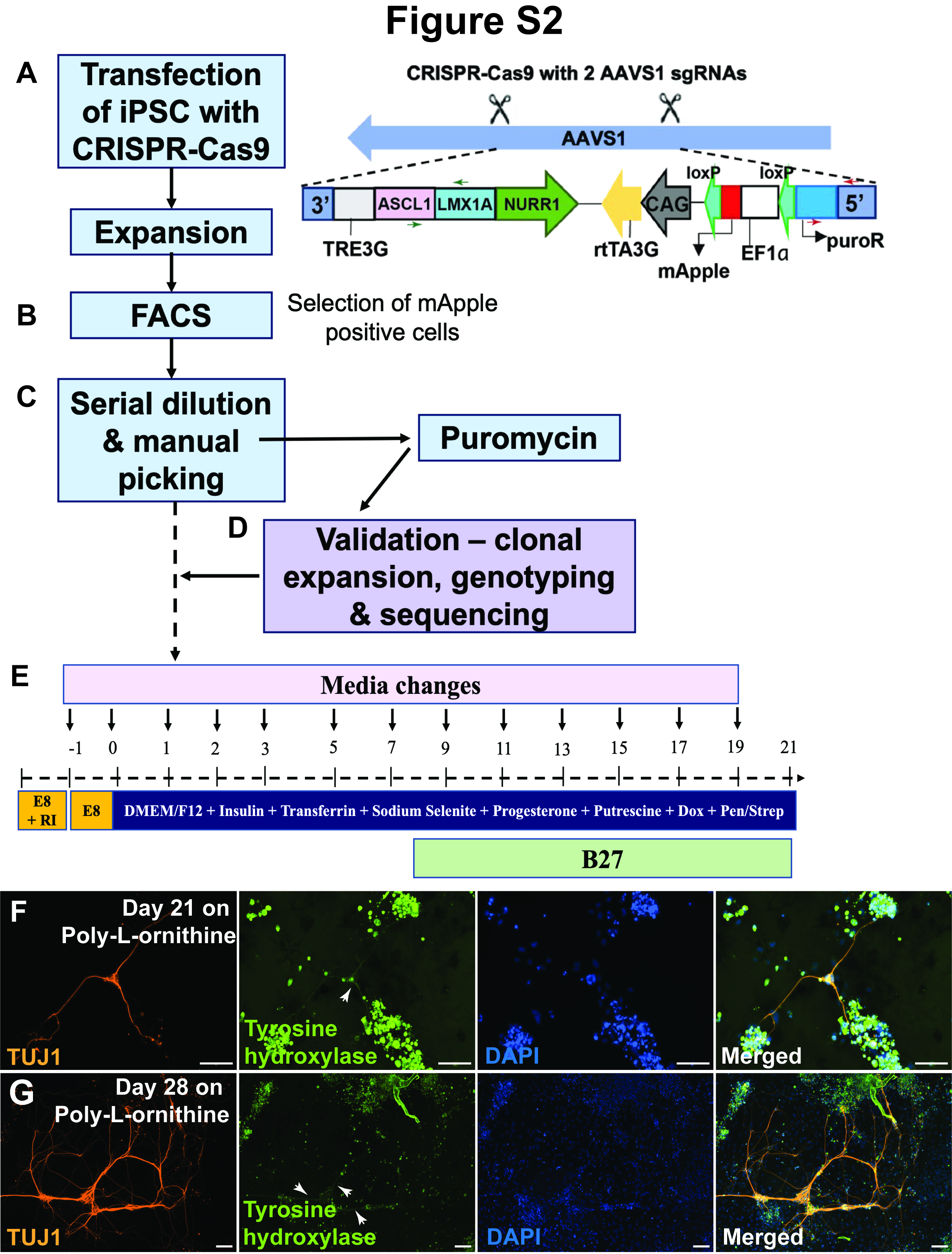

Supplement: Supplementary file 1 [file cells-13-01016-s001.zip › Figure S2.tif]

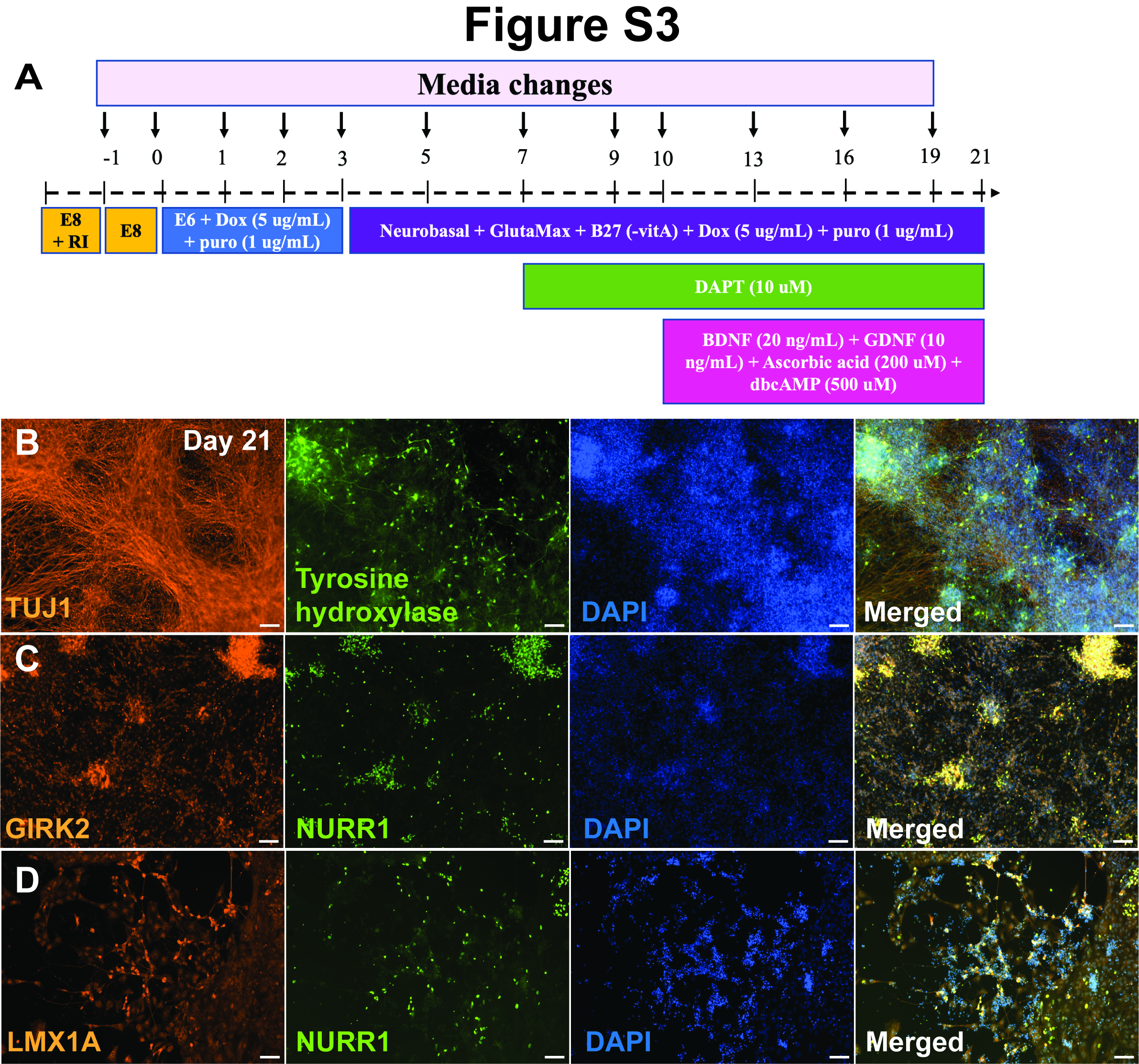

Supplement: Supplementary file 1 [file cells-13-01016-s001.zip › Figure S3.tif]

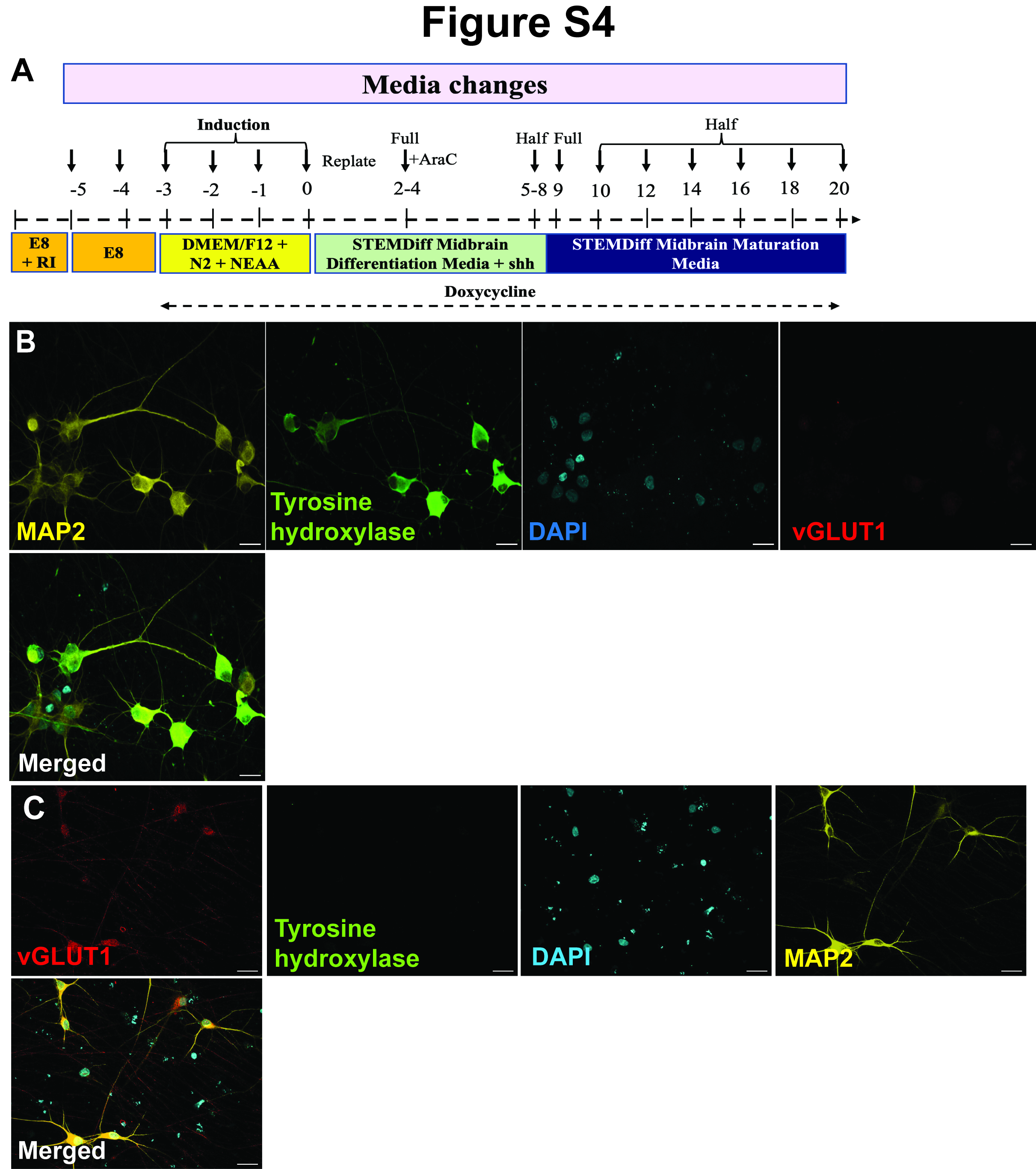

Supplement: Supplementary file 1 [file cells-13-01016-s001.zip › Figure S4.tif]
